# Idiopathic peripheral neuropathy increases fall risk in a population-based cohort study of older adults

**DOI:** 10.1186/1757-1146-5-S1-P19

**Published:** 2012-04-10

**Authors:** Jody L Riskowski, Lien Quach, Brad Manor, Hylton B Menz, Lewis A Lipsitz, Marian T Hannan

**Affiliations:** 1 Institute for Aging Research, Hebrew SeniorLife, Boston, Massachusetts 02131-1011, USA; 2 Harvard Medical School, Boston, Massachusetts 02115-6092, USA; 3 Gerontology and Interdisciplinary Medicine and Biotechnology, Beth Israel Deaconess Medical Center, Boston, Massachusetts 02115-6092, USA; 4 Musculoskeletal Research Centre, La Trobe University, Bundoora, Victoria 3086, Australia

## Background

Peripheral neuropathy (PN) is often associated with specific diseases; however, research suggests that idiopathic PN is prevalent in older adult populations [1]. Foot ulceration is the traditional medical concern with PN, but people with PN may also have disproportionately more falls [2]. Therefore, our objective was to evaluate associations between PN and prospectively-ascertained falls in older adults from the population-based MOBILIZE Boston Study.

## Materials and methods

Participants included 760 MOBILIZE Boston Study members. PN was assessed using Semmes-Weinstein monofilament testing [3], applying the modified Health ABC Study method [4,5]. Three PN status groups were defined: (i) no PN (referent), (ii) PN and known disease associated with PN (e.g., diabetes, autoimmune disease) (K-PN), and (iii) idiopathic PN (I-PN). Falls were tracked through monthly fall calendars over a mean 2.8 (range 1.4-4.3) year follow-up period. Unadjusted and adjusted (age, body mass index, physical activity, prior year fall, visual acuity, depression and number of medications) gender-specific negative binomial regression models determined associations between PN and falls.

## Results

**Figure 1:**
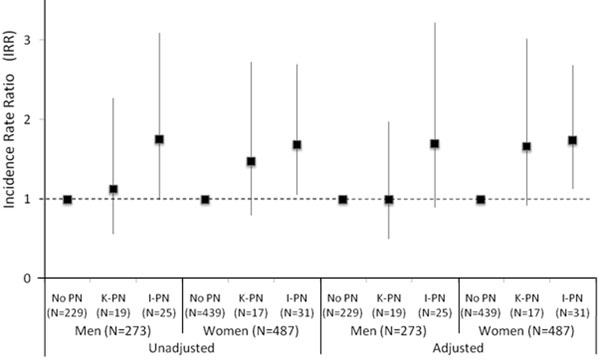
IRR between PN and falls

I-PN was associated with a higher fall incidence in men (incidence rate ratio [IRR] 1.76 [95% confidence interval 1.00–3.09]; Figure 1) and women (1.69 [1.06–2.70]). These higher IRRs with I-PN persisted even after covariate adjustment in women (1.68 [1.09–2.60]) and men (1.70 [0.90–3.22]), with men’s confidence interval widening. K-PN was not associated with an increased incidence of falling in men and had weak, non-significant effect in women.

## Conclusions

Idiopathic PN is an independent fall risk factor for women and men, suggesting that PN assessments should be included in fall risk evaluations. Future work to investigate mechanisms through which PN increases fall risk and to evaluate interventions that target fall risk in individuals with PN, such as insoles with low-grade vibrations [6], is needed.
